# Engineering tissue‐specific blood vessels

**DOI:** 10.1002/btm2.10139

**Published:** 2019-07-02

**Authors:** Lauren A. Herron, Corey S. Hansen, Hasan E. Abaci

**Affiliations:** ^1^ Department of Dermatology Columbia University Irving Medical Center New York NY 10032

**Keywords:** microphysiological systems, microvasculature, organ models, organ‐specific vasculature, vascular diversity, vascular heterogeneity, vascularized organs

## Abstract

Vascular diversity among organs has recently become widely recognized. Several studies using mouse and human fetal tissues revealed distinct characteristics of organ‐specific vasculature in molecular and functional levels. Thorough understanding of vascular heterogeneities in human adult tissues is significant for developing novel strategies for targeted drug delivery and tissue regeneration. Recent advancements in microfabrication techniques, biomaterials, and differentiation protocols allowed for incorporation of microvasculature into engineered organs. Such vascularized organ models represent physiologically relevant platforms that may offer innovative tools for dissecting the effects of the organ microenvironment on vascular development and expand our present knowledge on organ‐specific human vasculature. In this article, we provide an overview of the current structural and molecular evidence on microvascular diversity, bioengineering methods used to recapitulate the microenvironmental cues, and recent vascularized three‐dimensional organ models from the perspective of tissue‐specific vasculature.

## 1 INTRODUCTION

Vasculature connects all organs in the body and allows for the systemic interaction of the organs through soluble factors, nutrients, and cells. It is widely appreciated now that vasculature does not only function as a transport barrier or conduit, but it also regulates the homeostasis and tissue regeneration as a signaling tissue.^1^ Starting from early organogenesis, vasculature remains in direct contact and continuously interacts with the local microenvironment within each organ, leading to anatomical, molecular, and functional heterogeneities. Although anatomical characteristics of tissue‐specific vasculature have been well‐documented for centuries of research and observations, molecular and functional differences have only recently begun to be uncovered due to the recently available techniques permitting global gene expression analyses. The majority of these studies, so far, have been conducted with animals or human fetal tissues, while the differences in adult human vasculature and several organs are yet to be understood.^2^, ^3^, ^4^

In the last decade, significant progress has been made in the bioengineering of human vasculature from cultured primary cells and induced pluripotent stem cell (iPSC)‐derived vascular cells in parallel with the advancements in microfluidics and biomaterials fields that allowed for recapitulating the vascular microenvironment. Vascular tissue engineering has involved different approaches with incremental levels of complexity including monolayer cultures of endothelial cells (ECs) under shear‐stress and cyclic stretch,^5^ induction of angiogenesis in 3D hydrogels using ECs and other proximal cell types,^6^, ^7^, ^8^ such as smooth muscle cells (SMCs), iPSC‐derived vascular organoids,^9^ and three‐dimensional (3D) hydrogel‐based microfluidic models.^10^ The expertise gained in vascular tissue engineering has recently been employed to incorporate vasculature into 3D models of various other organs. The central motivation behind developing such models has, so far, been to mimic the vascular transport mechanisms within organs for drug testing and to promote the viability of tissue grafts. However, with the increasing knowledge in understanding vascular heterogeneity, these vascularized organ models may also represent invaluable platforms in the near future to dissect the roles of microenvironmental cues in the induction and maintenance of vascular heterogeneity in organs.

In this review, we compile anatomical and molecular evidence on microvascular diversity between organs, and discuss the recent vascularized 3D organ models from the perspective of tissue‐specific vasculature. We primarily focus on the blood vasculature and in vitro vascularization techniques rather than the lymphatic vasculature or in vivo grown blood vessels.

## 2 HETEROGENEITY OF BLOOD VESSELS IN DIFFERENT ORGANS

The heterogeneity of vasculature between organs at the anatomical level has been known for a long time. Recent studies have elucidated these differences and demonstrated that there are detectable and distinct structural and molecular differences that allow for the identification of organ‐specific vasculature.^2^, ^3^, ^11^ Other studies also showed that structural and molecular differences can exist within an organ depending on location of the vasculature.^3^, ^12^, ^13^ In this section, we will review and compare structural and molecular differences in the microvasculature of different organs as well as within the organs.

### 2.1 Structural heterogeneity

Kidney vasculature has some specific differences compared to other organ vasculature. The kidney microvasculature is fenestrated, comprising of ECs with pores 60–80 nm in diameter in order to facilitate diffusion.^12^ This type of fenestrated endothelium is usually seen if the organ is involved in filtration. In addition, there are specialized visceral epithelial cells with “feet” projections that wrap around specialized capillaries called podocytes that leave slits to allow blood to be filtered. Podocytes specifically wrap around capillaries in the glomerulus and are fused to the basement membrane (BM) of vasculature, which is the extracellular matrix (ECM) that separates the endothelium from other cells.^12^, ^14^ The BM of kidney endothelium is thicker than most other organs and densely vascularized.

Similar to the kidney, the liver also has a discontinuous and highly permeable endothelium that allows for efficient diffusion and filtration.^12^ The liver endothelium possesses a disorganized BM and has unsealed intracellular clefts and specific sinusoidal endothelium. A liver sinusoid is a specialized capillary where the blood supplies from the portal vein and hepatic arteries meet. Sinusoids lack the diaphragm that most other capillaries have to cover pores and instead just contain open pores.^15^ The sinusoidal endothelium is very permeable and has the highest rates of endocytosis in the human body. Liver endothelium is surrounded by special liver specific macrophages called Kupffer cells.^15^ Liver vasculature has origins in endocardiac cells^16^, ^17^ and some literature suggests that the liver and its vasculature play a role in early organogenesis.^11^, ^18^ The liver is one of the organs that contains multiple vasculature systems. It has two separate vasculature systems, portal and arterial. The portal vasculature carries lipid rich, poorly oxygenated blood from the intestine to the liver, and the arterial vasculature carries nutrition to the liver.^11^ Liver portal “veins” are not true veins because they do not empty directly into the heart and instead meet the nutrient rich blood of the arterial vasculature in the sinusoids. Liver ECs serve multiple purposes such as clearing waste and antigen presentation.^11^, ^19^ During development, they also promote organ formation before assuming vasculature function.^11^

The brain has one of the most well‐regulated vasculature surrounded by a complex microenvironment, which makes it difficult to replicate in vitro. The vasculature of the blood–brain barrier (BBB) contains tight junctions and sealed intracellular clefts to prevent transcytosis. To meet the metabolic needs of the brain, the endothelium of brain vasculature contains more mitochondria than the endothelium of other organs.^20^ Within the vasculature, there is an established polarity between the luminal and abluminal membranes. The BBB has endothelial and parenchymal BMs.^21^ The endothelial BM surrounds the endothelium, which is then surrounded by pericytes, which is surrounded by the parenchymal BM. Astrocytes attach to the parenchymal BM.^21^ There are multiple cell types surrounding the vasculature including astrocytes and pericytes, as well as many ECM proteins such as collagen IV, proteoglycans, and laminins.^11^, ^22^ Astrocytes specifically are critical to brain vasculature development and structure, including tight junction formation.^11^ BBB ECs express specific omega‐3‐fatty acids. The BBB creates crucial obstacles to drug delivery due to its high regulation and low rates of transcytosis and has been studied in depth for characterization and drug delivery purposes. A comprehensive recent review on BBB can be found elsewhere.^23^

Gut vasculature has similarities to brain vasculature, specifically the BBB. The gut vasculature barrier also contains tight junctions and adherens junctions, as well as pericytes and glial cells closely interacting with the endothelium.^24^ Intestinal ECs are surrounded by glial fibrillary acidic protein (GFAP)‐positive glial cells. GFAP is known to be involved in maintaining cell structure and cytoskeleton and seen most commonly in a variety of cells in the central nervous system.^25^ The glial cells surrounding intestinal ECs do not emit action potentials like neuroglia but can communicate using Ca^2+^ signals. They also share similar morphology to astrocytes. Gut endothelium is also fenestrated. Gut vasculature allows for diffusion of larger molecules than BBB, allowing molecules as big as 4 kDa to diffuse, significantly larger than those that can diffuse across the BBB (<500 Da).^24^

Bone vasculature contains discontinuous epithelium as well. The capillaries are organized densely into columns near growth plates.^11^, ^26^ Capillaries form branched structures in bone marrow. Capillaries are dense near growth plates to control osteoprogenitors, and are branched in marrow for contact with hematopoietic cells with less permeable arteries in marrow to maintain stem cells. Bone also contains sinusoidal endothelium, which is not surrounded by pericytes but is instead surrounded by special myeloid cell clusters.^27^ There are two different types of vessels, H and L, with distinct metabolic environments; arteries feed into high velocity (H) vessels, which then feed into low velocity (L) vessels. H vessels have high oxygen content and L vessels have low oxygen content.^11^ A recent study has identified a previously unknown type of blood vessel that are seen in bones called trans‐cortical vessels.^28^ They cross through the bone and connect in the periosteum. The exact functions are unknown but some proposed functions are immune cell transport, nutrient transport for osteocytes within bones, hematopoiesis, and connecting osteocytes and osteoclasts.^28^, ^29^, ^30^

Skin vasculature extends horizontally through the dermis and through the dermal‐subcutaneous junction. The capillaries form loops throughout the dermis near the hair follicles, where the dermal papillae are located.^31^ The microvasculature has a homogeneous and organized BM. The ECs are surrounded by SMCs and elastin fibers which are all encased by the BM. Pericytes are found surrounding the ECs and assist in the formation of tight junctions; they connect to the ECs through the BM.^31^ The vascular walls of dermal capillaries are wider than they are in other organs, with the average being 0.5–1 μm wide, whereas in other organs they are smaller at approximately 0.1 μm.^31^ Capillaries in the lower third of the dermis can be twice as large than the capillaries in the rest of the dermis due to the role those vessels play in the inflammatory response. Skin endothelium is fenestrated only near sweat glands and dermal papillae. Some of the arterioles and venules are directly linked and at these connections, there are glomus bodies, which are smooth‐muscle like cells that help shunt blood away from skin when exposed to cold.^32^

The heart has multiple types of endothelium. There is the endocardium endothelium, valvular endothelium, coronary endothelium, and myocardial microvessel endothelium.^12^ The endocardium possesses larger ECs than the other forms of endothelium. It also contains microvilli, abundant gap junctions, and deeper intracellular clefts containing a couple of tight junctions. The endocardium has higher concentrations of von Willebrand Factor than other parts of the heart.^3^ During cardiac development, the ECs that make up valves undergo delamination and lose cell–cell contacts to form heart valves. The delaminated cells later invade the ECM. The ECs that compose the valves have differing gene expression depending on what side of the valve the ECs are on.^12^ Coronary vessels, which are the vessels that supply blood to the myocardium, refers to coronary arteries, which supply the heart with oxygenated blood, and cardiac vessels, which drain the oxygen poor blood for oxygenation. The coronary vessels are epicardial, meaning they run along the surface of the heart. There is a greater concentration of SMCs in the coronary vessels than there is in any others excluding the femoral artery. The myocardial microvessels contain a high number of ECs, with the number of cells being approximately three times greater than that of the myocardial cells. Myocardial capillaries contain continuous endothelium and little to no gap junctions.

The lung, similar to the liver, has two different vasculature systems. Lung endothelium is continuous. There is the pulmonary system and the bronchial system. The pulmonary system is high volume but low pressure, and the bronchial system is high pressure and low volume. The pulmonary system is responsible for gas exchange whereas the bronchial system is responsible for delivering oxygen (O_2_) to the bronchial tree. Throughout the lungs, in the pulmonary system, capillaries form a dense net‐like structure with alveoli forming the holes, through which blood seeps through.^12^ Gas exchange occurs at the air‐blood barrier, which is composed of endothelium and epithelium separated by a very thin BM, of 0.1 μm. Bronchial ECs leak more and have higher angiogenic capability than pulmonary ECs. The microvasculature of the lung preferentially binds Griffonia simplicifolia lectin over macrovasculature allows for differentiation using staining.^12^

### 2.2 Molecular heterogeneity

Although there are a large number of papers discussing the structural differences of endothelium between organs, there are significantly fewer studying the molecular differences such as the surface and angiogenic markers, and even fewer papers studying human specific markers. This disparity is due in part to only recent availability of high throughput gene profiling techniques, such as RNA sequencing and limitations in obtaining human organs.

A landmark paper demonstrating EC heterogeneity by Chi et al.^4^ showed there were genetic differences between the ECs of arteries and veins, as well as between large vessels and microvessels. Using cultured human ECs, the paper also showed differences between ECs depending on the organ where the cells originated. While limited at the time with the gene expression analysis and the tissues available, this article provided a very important foundation that was able to be expanded upon by Marcu et al.^3^ and Nolan et al.^2^

**Figure 1:**
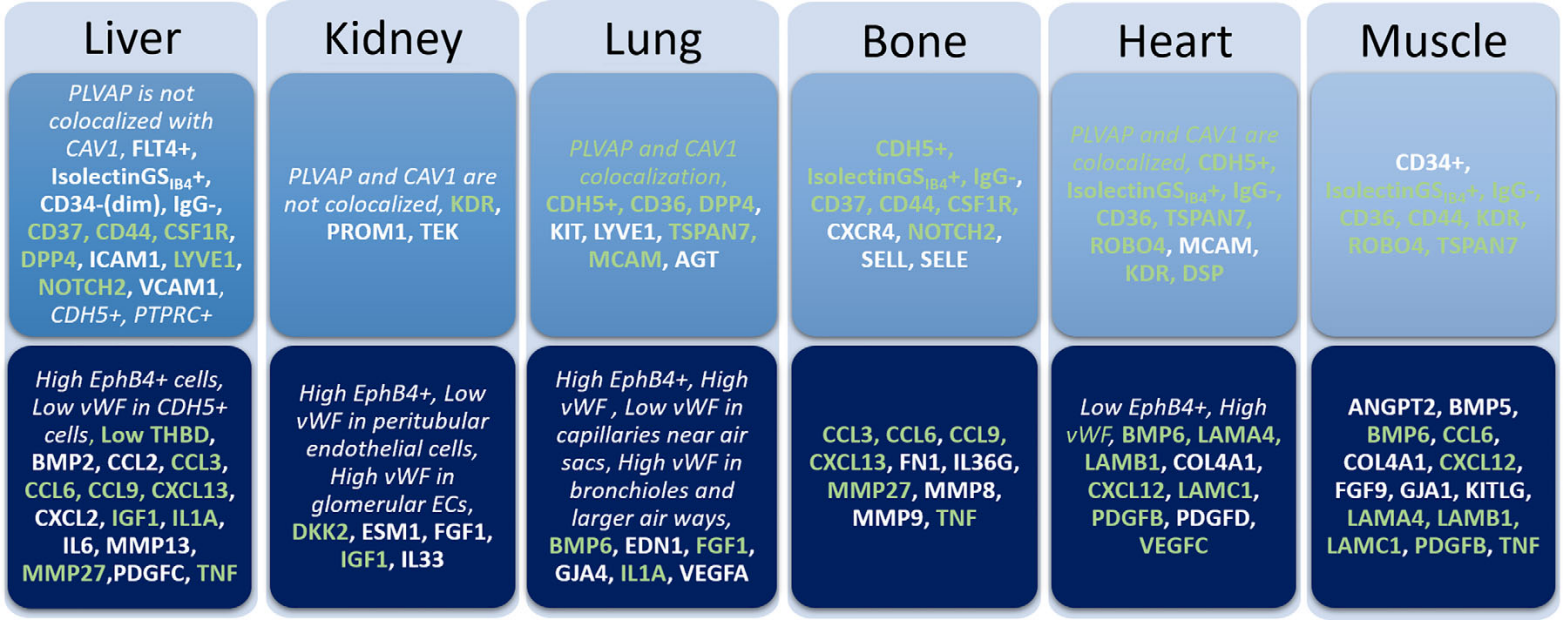
Compilation of organ‐specific angiogenic markers and endothelial markers. Green text indicates shared marker with another organ. Bold text indicates the marker is from Nolan et al.^2^ and italic text indicates the marker is from Marcu et al.^3^ All gene abbreviations follow the internationally approved HUGO gene nomenclature

Up to this point, most of our knowledge on organ‐specific vasculature is based on mouse experiments due to the much greater access to mice in different stages of development than to human fetal tissue. The information in Figure 1 is compiled from Marcu et al. and Nolan et al., both landmark papers detailing critical molecular differences between the vasculature of different organs. Nolan et al.^2^ identified angiogenic and molecular markers of the ECs collected from different organs of mice, whereas Marcu et al.^3^ used 3‐month‐old human fetuses which provides one of the only in‐depth studies on organ specific gene expression in developing human vasculature, allowing for a more relevant study into the heterogeneity of ECs. Figure 1 displays that there is a diversity of gene expression in the ECs from the vasculature of different organs. Although there is overlapping gene expression between organs, there are sufficient differences between the organs listed that it could be possible to differentiate the individual organ's ECs, and thus the vasculature, from each other.

Although not specified in Figure 1, within some organs the vasculature expresses different surface markers and angiogenic markers depending on where the vasculature is located. The heart, kidney, liver, and lungs are all organs with differing gene expression in ECs depending on the location of the cells. Within the heart alone, there is a different in the localization of certain proteins, such as endothelial nitric oxide synthase 3 (eNOS).^12^ Within the endocardium, eNOS is found in the golgi, but in the ECs of the cardiac capillaries, it is found spread throughout the cytoplasm. Endocardial ECs express Cx37, Cx40, and Cx43, which are connexin proteins that indicate the absence of gap junctions; however, none of these proteins are expressed in the ECs of the cardiac capillaries.

Although both studies provide a lot of insight into vascular heterogeneity, something that must be considered is the different sources of the primary tissues. The different stages of development, the mice being neonatal and the human tissue coming from 3‐month‐old fetuses could have an effect on the gene expression patterns. Endothelial gene expression also differs due to aging, meaning there could be different gene expression in the ECs of vasculature harvested from adult organs compared to that from the 3‐month‐old human fetuses as well as from the newborn mice. in vitro vascularized organs can help to address those differences. Using current methods including microfluidic systems, 3D reconstructed models, or organoids, we can more accurately mimic the physical and chemical environment of the ECs to better understand the heterogeneity as well as attempt to maintain gene expression, which has been seen to change drastically after primary cells are harvested and cultured regularly for a small number of passages.

## 3 MIMICKING PHYSICAL AND CHEMICAL CUES IN VASCULAR MICROENVIRONMENT

The cellular function in the body is intricately regulated by the microenvironment through mechanical and chemical cues. When cells are removed from their host microenvironment and cultured in vitro, they lose their distinct cellular characteristics.^3^ For instance, ECs from different origins or organs, such as artery versus vein or brain versus kidney, lose the majority of their gene expression signature after several passages in culture and become phenotypically similar to each other, however some differences in their gene profiles may remain.^2^, ^3^, ^4^ For the effective use of these cells in tissue or disease modeling, or in regenerative medicine, physiologically relevant function of the cells must be restored by recreating the mechanical and chemical cues in the native microenvironment.

### 3.1 Mechanical cues

Vascular cells are constantly exposed to various physical stimuli such as shear stress, cyclic strain as well as different flow regimes (e.g., laminar or turbulent), and patterns (e.g., pulsatile or nonpulsatile).^33^ Cells can sense and transduce these mechanical cues to molecular signals. Historically, shear‐stress and cyclic strain exerted by blood flow were first recreated in vitro using large‐scale perfusion culture systems,^5^ including parallel plate flow systems and cyclic‐stretch devices, which later transformed into microfluidic systems containing microchannels typically made of plastics such as Polydimethylsiloxane (PDMS) or polycarbonate.^34^, ^35^, ^36^ More recently, microchannels were created in 3D reconstructed ECM and hydrogels that permitted studying the permeability of molecules through the endothelial barrier and for incorporating other vascular cell types (e.g., smooth muscle cells), paving the way for generating high‐fidelity microvascular tissues.^6^, ^37^ The surrounding ECM in these models can also provide mechanical signals, which affect various cellular responses responsible for homeostasis and angiogenesis.^38^ Employing a similar hydrogel approach, Qiu et al. recently developed a gelatin‐agarose‐based microfluidic system that enabled long‐term maintenance of blood vessels up to 2 months under physiological shear stress.^10^

The blood flow in microfluidic platforms is typically mimicked by perfusing culture medium by peristaltic pumps, integrated micropumps, or centrifugal microfluidics. As an alternative and simple strategy, the Shuler's Group introduced a recirculating gravity‐driven approach eliminating the need for external pumps and tubing.^39^ One major drawback of this approach was that the medium circulating between two reservoirs caused bidirectional flow and reciprocating shear stress, which may induce a diseased phenotype in ECs. Therefore, previous studies adapting this approach either had to evade the inclusion of vasculature in their system^40^ or minimize the shear stress on ECs^41^ to prevent the undesired effects of bidirectional flow. A recent progress from the same group^42^ circumvented this limitation by adding supporting channels and passive valves into their microfluidic platform to generate a recirculating unidirectional gravity‐driven flow, removing the biggest obstacle in the way of using this elegant pumpless system.

### 3.2 Chemical cues

The vascular microenvironment contains many cytokines, chemokines, and growth factors as well as ECM components. These are all regulated by multiple cell types comprising or surrounding the blood vessels. Current vasculature models and culture methods mimic these chemical cues around ECs through: (a) adding soluble factors directly into culture medium, (b) controlling nutrient and oxygen levels, or (c) coculture of ECs with other cell types, such as SMCs, pericytes, or fibroblasts. Typically, growth factors such as Vascular endothelial growth factor (VEGF) and basic fibroblast growth factor are supplemented into culture medium to induce spontaneous capillary formation of ECs in hydrogels.^7^, ^43^ Despite its success in capillary formation, this method does not allow for maintaining the function or stability of the capillaries. In such studies, capillaries may regress soon after their formation or can be leaky, and require additional stabilizing factors including Transforming growth factor beta, angiopoietin 1, and Insulin‐like growth factor,^44^ relying on a delicate balance between angiogenic and stabilizing factors. To establish a stable vasculature with an enhanced endothelial barrier function, ECs were cocultured in hydrogels with other cells types that are physiologically proximal to ECs, such as pericytes, astrocytes, and dermal fibroblasts.^45^, ^46^ Smooth muscle cells and pericytes can stabilize the microvessels through angiogenic factors and direct cell–cell contact.^45^, ^46^ Astrocytes have been used to improve the endothelial barrier function in BBB models.^47^ Dermal fibroblast not only modifies the surrounding ECM but also secretes stabilizing factors to stabilize de novo microvessels.^45^, ^46^

Oxygen concentration is an important physiochemical factor regulating vascular responses throughout the body. Dissolved oxygen levels in vasculature vary in a broad range of 5% to 12% O_2_ in physiological conditions and can go below 1% O_2_ during ischemic injury and in tumors. When vascular cells are cultured in conventional culture systems, they are exposed to atmospheric oxygen (21% O_2_), or uncontrolled fluctuations in dissolved oxygen due to oxygen consumption by cells.^48^ Vascular cell types can sense and adapt to these changes in oxygen levels through different mechanisms exhibiting various responses including angiogenesis, proliferation, apoptosis, and migration. Microfluidic culture platforms developed using different approaches and design principles helped circumvent this problem by finely tuning the oxygen concentration at the cellular level and allowed for recapitulation of healthy and diseased conditions.^34^, ^49^

These advancements on recreating the vascular milieu in vitro allow for restoring the physiological relevance of vascular cells in culture. Notably, some of the physical and chemical cues discussed above also show variations between organs. For example, the average levels of shear stress and oxygen tension may significantly vary depending on the organ and localization within organs.^33^ Therefore, factoring in these tissue‐specific and spatial variations may be a good strategy for future studies to improve the tissue relevance of the vascular models. In light of the progress made in recapitulating the vascular microenvironment, several studies recently accomplished the integration of vascular tissues into engineered organs, representing a significant step towards developing tissue‐specific vasculature models. We discuss some of the pioneering examples of this emerging field in the next section.

## 4 IN VITRO MODELS OF VASCULARIZED ORGANS

In the past decade, there has been a growing interest and major progress in engineering physiological human tissues for regenerative medicine and in developing microphysiological systems for human‐relevant disease modeling and drug development. Having blood vessels in engineered tissues is crucial for the viability of tissue grafts as well as for mimicking the systemic delivery/clearance of drugs or cells to/from organs. These two aspects, so far, have been the main driving force for incorporating the vascular component into engineered organs. However, in light of the growing evidence on microvascular heterogeneity throughout the body, now these vascularized organ platforms can also shed light on tissue‐specificity of human vasculature.

There are three main engineering approaches to build human tissues in vitro: (a) 3D reconstructed models, in which cells are embedded and cocultured in 3D hydrogels or decellularized ECM, (b) microfluidic models, in which cells or tissues are cultured in individual chamber connected with microchannels and/or porous membranes, and (c) self‐organized tissue models, in which primary or pluripotent cells are assembled in spheroids and cultured or stimulated for their self‐organization. In this section, we will provide an overview of the emerging vascularized organ models achieved through these main approaches and compare the engineered vasculature in the context of organ‐relevance, as summarized in Table 1.

**Table 1:** Summary of vascularized organ models categorized in Section 4

Organ	Organ model	Vascularization method	Perfusion	Cell types	Confirmation assays	Citation
Bone	Reconstructed	Spontaneous	In vitro	hMSCs, HUVECs	CD31 immunostaining	Correia et al.^50^
	Reconstructed	Spontaneous	In vitro	hMSCs, ECs	CD31 immunostaining	Marturano‐Kruik et al.^51^
	Reconstructed	Spontaneous	No	hASCs, HDMECs	CD31 immunostaining	Wenz et al.^52^
	Reconstructed	In vivo transplantation	No	Rat MSCs	Alkaline phosphatase, H&E	Kawamura et al.^53^
	Reconstructed	In vivo transplantation	In vivo	Rabbit MSCs	H&E	Wang et al.^54^
Heart	Reconstructed	Prepatterned	In vitro, in vivo	hMSCs, HUVECs, hESC‐CMs	CD31 immunostaining, Masson's Trichrome	Zhang et al.^55^
	Self‐organized	Spontaneous	No	ESC‐ and iPSC‐derived axial and ventricular CMs, cardiac fibroblast	CD31, Cx43 immunostaining	Zhao et al.^56^
	Self‐organized	Spontaneous	No	Mouse EC^iLacZ^	X‐gal staining	Stoehr et al.^57^
	Self‐organized	Prepatterned	No	Rat CMs, ECs	Cx43 immunostaining	Fleischer et al.^58^
	Self‐organized	Spontaneous	In vivo	HUVECs, iPSC‐CMs	CD31 immunostaining	Arai et al.^59^
	Self‐organized	Spontaneous	In vivo	HUVECs, human aortic smooth muscle cells	CD31 immunostaining, Masson's Trichrome	Itoh et al.^60^
	Self‐organized	Spontaneous	No	hCM, human coronary artery ECs, iCF	CD31 immunostaining	Polonchuk et al.^61^
	Microfluidic	Spontaneous	No	Human cardiac ECs	VECad+, vWF, PV1/CAV1 Immunostaining, TEM	Marcu et al.^3^
Brain	Reconstructed	Spontaneous	No	ESC‐derived ECs, NPCs, MSCs, and microglia/macrophage precursors	CD31 immunostaining	Schwartz et al.^62^
	Self‐organized	Spontaneous	No	iPSC‐derived ECs	CD31 immunostaining	Pham et al.^63^
	Self‐organized	Spontaneous	In vivo	hESCs, host vascularized	CD31, blood immunostaining	Mansour et al.^64^
	Microfluidic	Prepatterned	In vitro	Human iPSC‐derived brain microvascular ECs	Claudin‐5, ZO1 immunostaining	Wang et al.^41^
	Microfluidic	Prepatterned	In vitro	hiPSC‐ECs, hES‐derived neuronal stem cells, human motor neuron progenitors	iPSC‐EC actin, MN tubulin immunostaining	Osaki et al.^65^
	Microfluidic	Prepatterned	In vitro	hiPSC‐ECs, hPCs, hACs	CD31 immunostaining	Campisi et al.^66^
	Microfluidic	Prepatterned	In vitro	iPSC‐dhBMECs, HUVECs	ZO1, occluding, claudin‐5, GLUT1, PGP immunostaining	Linville et al.^67^
Liver	Self‐organized	Spontaneous	In vivo	iPSC‐HEs, hMSCs, HUVECs	CD31 immunostaining	Takebe et al.^68^
	Self‐organized	Spontaneous	In vivo	mCherry iPSC‐ECs, HUVECs, BMSCs, iPSC‐derived hepatic endoderm cells	CD31 immunostaining	Takebe et al.^69^
	Self‐organized	Spontaneous	In vivo	Hepatic endoderm, ECs, MCs	CD31 immunostaining, scRNA‐seq	Camp et al.^70^
	Self‐organized	Spontaneous	In vivo	NHDFs, HUVECs, hepatocytes	CD31 immunostaining, H&E	Sasaki et al.^71^
	Microfluidic	Prepatterned	In vitro	Human hepatocytes, stellate cells, Kupffer cells, LSECs, and porcine LECM	FITC‐albumin transport measurement, CellTracker	Li et al.^72^
	Self‐organized	Spontaneous	No	Stellate cells, LSECs, hMSCs, hUCBSCs	CD31 immunostaining	Li et al.^73^
	Microfluidic	Spontaneous	No	Human hepatic ECs	VECad+, vWF, PV1/CAV1 immunostaining, TEM	Marcu et al.^3^
Skin	Self‐organized	Spontaneous	No	NHKs, NHFs, hMVECs	CD31 immunostaining	Supp et al.^74^
	Self‐organized	Spontaneous	In vivo	hLECs, NHFs, hDMECs	CD31 immunostaining	Marino et al.^75^
	Self‐organized	Spontaneous	In vivo	hMVECs, HUVECs, NHKs, NHFs	CD31 immunostaining, Masson's Trichrome	Gibot et al.^76^
	Reconstructed	Prepatterned	In vitro	hEK, hDF, hDMEC	CD31 immunostaining	Groeber et al.^77^
	Reconstructed	Prepatterned	In vitro, in vivo	NHFs, NHKs, iPSC‐derived ECs	CD31 immunostaining	Abaci et al.^78^
Lung	Self‐organized	Spontaneous	No	Human lung epithelial cells, hLMECs, hLMCs	CD31 immunostaining	Tan et al.^79^
	Microfluidic	Spontaneous	No	Human lung ECs	VECad+, vWF, PV1/CAV1 immunostaining, TEM	Marcu et al.^3^
Gut	Reconstructed and self‐organized	Prepatterned	In vitro	HUVECs, epithelial cells	Lucifer yellow permeability assay, VE‐cadherin staining, ZO‐1 staining	Kitano et al.^80^
	Microfluidic	Prepatterned	In vitro	HIMECs, epithelial cells (intestinal crypt)	RFP labeled HUVECs, CD31 staining	Kasendra et al.^81^
Kidney	Self‐organized	Spontaneous	In vitro, in vivo	MSCs, HUVECs, unspecified renal cells	CD31, FITC‐dextran infusion	Takebe et al.^82^
	Self‐organized	Spontaneous	In vivo	HUVECs, MSCs, hIPSC‐derived nephron progenitor spheres	Nephrin staining, GFP staining	Sharmin et al.^83^
	Microfluidic	Spontaneous	No	Human renal ECs	VECad+, vWF, PV1/CAV1 Immunostaining, TEM	Marcu et al.^3^
Pancreas	Microfluidic	Prepatterned	In vitro	Whole islet	CD31 immunolabeling, fluorescent dextran	Sankar et al.^84^
	Self‐organized	Spontaneous	In vivo	Islets with HUVECs ± adMSCs	H&E staining, Griffonia simplicifolia lectin‐1 staining	Vlahos et al.^85^
Skeletal muscle	Self‐organized	Spontaneous	In vivo	Myogenic progenitors, ECs, pericytes, motor neurons, neural progenitor cells	Human LAMIN A/C staining, CD31 staining, Western blot, isolectin staining	Maffioletti et al.^86^
	Reconstructed	Spontaneous	No	Muscle harvested from mice, ECs	H&E staining, RT‐qPCR	Carosio et al.^87^
Tumor	Self‐organized	Spontaneous	In vitro, in vivo	Tumor cells and ECs	EGFP+ transduction of ECs, confocal microscopy	Ehsan et al.^88^
	Microfluidic	Prepatterned	In vitro	Microdissected patient tumors and mouse xenograft tumors	Annexin V staining, 7AAD staining	Astolfi et al.^89^
	Microfluidic	Spontaneous	In vitro	ECs, cancer cells, fibroblasts, ECM	mCherry transduction of ECs, GFP transduction of GFP	Sobrino et al.^90^

Abbreviations: ECs, endothelial cells; ECM, extracellular matrix; hASCs, human adipose stem cells; HDMECs, human dermal microvascular endothelial cells; HE, hepatic endoderm; hLECs, human lymphatic endothelial cells; hMSCs, human mesenchymal stem cells; hUCBSCs, human umbilical cord blood stem cells; HUVECs, human umbilical endothelial vein endothelial cells; iPSCs, induced pluripotent stem cells; LSECs, liver sinusoidal endothelial cells; MCs, mesenchymal cells; MN, motor neuron; TEM, Transmission electron microscopy; NPC, neural progenitor cell; LECM, liver sinusoidal endothelial cells; NHK, Normal human keratinocyte; NHF, Normal human fibroblast; hMVEC, human microvascular endothelial cell; hEK,human embryonic kidney; hDF, human dermal fibroblast.

### 4.1 Bone

Bones perform many essential biological functions for an organism in addition to providing the physical structure, which supports and protects the organs. They are the source of blood cells and are infiltrated by an elaborate network of vasculature that allows the blood cells to migrate throughout the body. Therefore, bone vasculature is not only responsible for nutrient supply to the tissue, but also acts as the source of hematopoietic progenitor cells to replenish blood cells and to support vasculogenesis in different sites of the body.

There are two main approaches that have been used so far to engineer vascularized bone tissue. Bone models have been made through the reconstructed model method using decellularized bovine bones or bioprinted hydrogels such as chitosan, gellan gum, and gelatin‐methacrylol as a scaffold, which are then repopulated by different combinations of human mesenchymal stem cells (hMSCs), osteoblasts, and human umbilical endothelial vein ECs (HUVECs).^50^, ^91^

**Figure 2:**
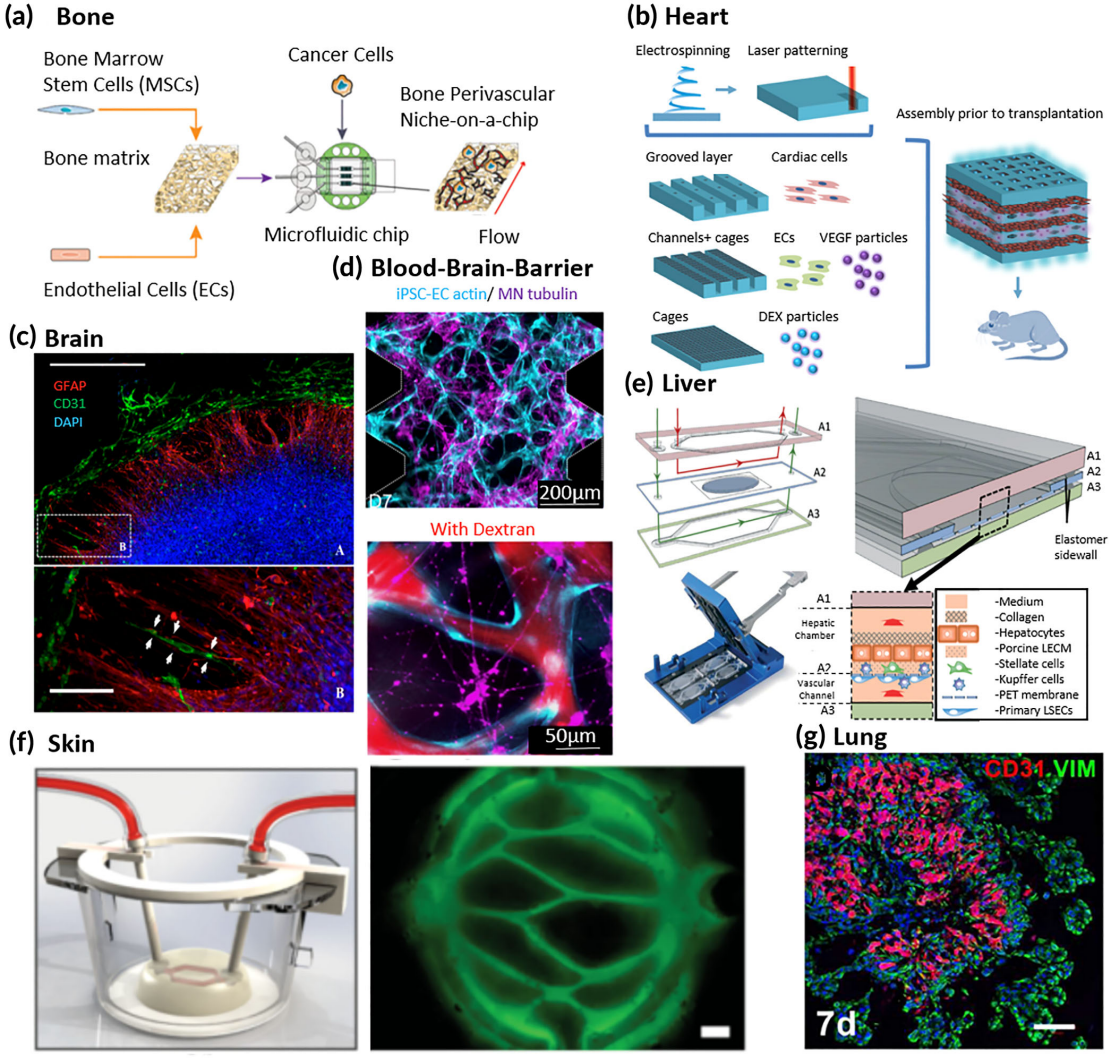
Current vascularized tissue engineered models. (a) Bone perivascular niche‐on‐a‐chip schematic displaying formation, usage, and flow direction.^51^ (b) Schematic depicting the construction of a thick cardiac patch.^58^ (c) Neural construct displaying endothelial cells aligning with glial cells.^62^ (d) A blood–brain‐barrier model displaying human‐induced pluripotent stem endothelial cells and motor neuron networks with dextran perfusion.^65^ (e) The vascularized liver acinus microphysiology system showing the various layers and location of liver sinusoidal endothelial cells.^72^ (f) Three‐dimensional rendering of blood vessel perfusion in skin constructs with immunofluorescence staining depicting physiological orientation of the blood vessels.^78^ (g) Airway organoid displaying vasculature in the center with airbuds on the outside of the organoid^79^

Vascularization of bone has been achieved by Vunjak‐Novakovic's group by coculturing hMSCs and HUVECs within a reconstructed bone scaffold. Growth factors were sequentially added and induced stable vasculature and osteogenic differentiation.^50^ They found that vascular development is improved when it is induced prior to osteogenesis and that the hMSCs during the osteogenic induction stage improve the outcome of the tissue. In addition, the same group has developed a bone perivascular (BoPV) niche‐on‐a‐chip to study early metastatic events arising in bone^51^ (Figure 2a). It has precisely controllable flow and the ability to test immunotherapy possibilities by introducing immune cells and tracking and quantifying their activity.

In another study, Wenz et al. cocultured human adipose stem cells (hASCs) and human dermal microvascular ECs (hDMECs) were combined with methacryloyl‐modified gelatin (GM) to form reconstructed 3D scaffolds. Softer GM gels were combined with HDMECs and hASCs and then indirectly cocultured with stiffer GM gels that had hASCs embedded. They found that the stiffer gels expressed bone‐specific proteins and the HDMEC gels formed capillary‐like networks after 14 days.^52^

In addition to in vitro models, some studies focused on prevascularization of bone grafts and implants. Kawamura et al. cultured rat bone marrow MSCs in interconnected porous hydroxyapatite ceramics to prevascularize the implants. The vascularized implants were confirmed through alkaline phosphatase staining and had de novo bone formation after 4 weeks of being implanted. In contrast, the nonvascularized implants was surrounded by necrotic tissue.^53^ Similarly, Wang et al. loaded MSCs into B‐tricalcium phosphate reconstructed ceramic scaffolds and implanted them into the femurs of rabbits. They found that there was more bone formation, more vascular sections, and higher VEGF expression in the prevascularized scaffolds compared to the nonvascularized scaffolds.^54^

### 4.2 Heart

The heart has the important job of pumping blood throughout the entire body, but it itself is highly vascularized. 2D culture is limited in its recapitulation of in vivo cardiac function due to the necessity of including multiple cell types, limited interaction with the ECM and between cells, and the inability to have distinct chambers.

A common hurdle for tissue engineering using iPSC‐derived cells is that the newly created models are immature compared to their in vivo counterparts. Ronaldson‐Bouchard et al. has addressed this problem for cardiac tissue through electrical stimulation.^92^ Specifically, for cardiac models, contractility must be able to replicate endogenous rhythm patterns and display similar action potentials.^93^

The AngioChip is a reconstructed model developed by the Radisic group that contains nanopores and micro‐holes to allow the formation of a vascular network.^55^ They have created vascularized cardiac and hepatic tissues with the cardiac model being successfully implanted in vivo with direct surgical anastomosis. In addition, they have also developed the Biowire II platform which can show electrophysiologically distinct atrial and ventricular tissues.^56^

Engineered heart tissue is a self‐organized model technique developed by Eschenhagen et al. and recently has had vascularization spontaneously form with a mix of unpurified cells from newborn mice hearts.^57^, ^94^ EC formation was found through tamoxifen administration with X‐gal staining for visualization.

Fleischer et al. have made a self‐organized model utilizing an electrospinning technique to create albumin fiber scaffolds with each layer having one of three structures^58^ (Figure 2b). After being electrospun, each layer is laser patterned to have either grooves, microtunnels and cages, or cages alone. In the microtunnels and cages layer, they incorporated double‐emulsion poly(lactic‐co‐glycolic acid microparticles that provide VEGF release over‐time to the ECs in the microtunnels to promote angiogenesis. To combine each layer to form a patch, they developed a thermoresponsive ECM‐based hydrogel that solidifies at 37°C. The 5 mm‐thick cardiac patches were transplanted into rats with and without VEGF particles. After 2 weeks, it was clear that the VEGF‐containing patches had a clear redness indicating the vessels were filled with blood, were nearly twice the blood vessel density, and occupied more than double the area of the patch percentage wise.

The Nakayama group utilized a 3D bio‐printer to create a self‐organized model using scaffold‐free tubular cardiac constructs.^59^ Cardiac spheroids were formed with a 50:25:25 mixture of iPSC‐derived cardiomyocytes (iCells), HUVECs, and Normal human dermal fibroblast (NHDFs), cultured for 7 days, and then 3D printed into a needle array to form a tubular structure. Contraction was measured by an in‐house method through fractional area changes recorded by video. The only vascular marker identified was CD31 through hematoxylin and eosin (H&E) staining with microvascular‐like formation. The Nakayama group has experience transplanting scaffold‐free tubular tissues into rats for in vivo study.^60^

Vascularized cardiac spheroids have been made by combining human coronary artery ECs, iPSC‐derived cardiac fibroblasts, and human primary adult cardiomyocytes or iPSC‐derived CMs at ratios simulating in vivo.^61^ The ECs formed a vascular network that was identified by staining for CD31 and found that CFs provide support to the vascular network formation by producing an ECM scaffold for single ECs.

### 4.3 Brain

One of the most complex organs in the body currently has two different methods for generating tissue engineered models. There are brain organoids, which are generated from neuronal progenitors derived from stem cells that then self‐organize into constructs or rudimentary spheres (Figure 2c). The others are BBB models that attempt to replicate the highly selective nature of the vascular network found in the brain.

Pham et al. has combined nonvascularized brain organoids derived from iPSCs generated in vitro with iPSC‐derived ECs (iECs) from the same patient.^63^ The organoids were embedded into Matrigel containing 250,000 iECs and then transplanted into mice. Through CD31 Immunofluorescence (IF) staining, vasculature can be observed originating from the outside of the organoid and penetrating into the center. Mansour et al. generated brain organoids developed from hESCs and transplanted them into mice. They did not include human ECs before the transplant, but did find that perfused host vasculature invaded the graft between Day 5 and 14 postgraft.^64^

Using iPSC‐derived brain microvascular ECs (iPSC‐BMECs), Wang et al. has created a pumpless microfluidic platform. It contains integrated electrodes for transendothelial electrical resistance monitoring, has similar blood residence time compared to in vivo, and minimizes wall shear stress.^41^ Tight junctions were identified through immunofluorescent staining.

The Kamm group has developed many novel methods for BBB tissue engineering. They have combined motor neuron (MN) spheroids with HUVECs into both macro‐scale and microfluidic systems^65^ (Figure 2d). They found that bi‐directional signaling between the neuronal networks and vascular networks and their platform is a great model to gain a better understanding of these interactions. The MN spheres were cocultured with ECs after 31 days, during which they found the formation of MN and EC networks. To further advance their BBB model, the Kamm group tri‐cultured iPSC‐ECs, pericytes, and astrocytes.^66^

Linville et al. has created a BBB microfluidic model that resembles the postcapillary venules (PCVs).^67^ They used iPSC‐derived brain microvascular ECs (dhBMECs) and showed that they have similar cylindrical geometry, cell‐ECM interactions, and shear flow of in vivo human PCVs. Their diameter is relatively thick compared to other blood vessels at 150 μm. These dhBMECs were incorporated into a PDMS‐based microfluidic chip and was found to have similar restrictive permeability to PCV in rats.

### 4.4 Liver

The vascularization of engineered liver models is essential in recapitulating the drug metabolizing and detoxifying functions of the liver. Many diseases affect the liver such as hepatitis and cirrhosis, where liver transplantation is often required. There is generally a long wait list for patients requiring a transplant and graft rejection is common. An autologously generated liver tissue would greatly benefit these patients by avoiding immunosuppression and reducing the wait time to receive a transplant.

The Taniguchi group developed a self‐organized model by combining iPSC derived hepatic endoderm cells (iPSC‐HEs), HUVECs, and hMSCs in 2D culture and then after 48 hr noticed visible organized 3D cell clusters.^68^ These liver buds displayed vasculature intermixed throughout the entire organoid. They have further developed this technique by generating organoids entirely derived from iPSCs and developed a massive clinical‐scale manufacturing platform for drug screening.^69^ Using single cell RNA‐seq, Camp et al. have further characterized liver buds by identifying that they have a different gene expression profile when compared to 2D culture.^70^

A self‐organized method using a layer‐by‐layer cell coating technique is being developed by Sasaki et al. Hepatocytes were added to a coculture of HUVECs and NHDFs.^71^ Non‐obese diabetic/Severe combined immunodeficient mice were either grafted with liver tissue onto the subcutaneous space or injected subcutaneously with a hepatocyte suspension of 8 × 10^5^ cells. After 3 weeks, vasculature was found through histological analysis.

The Taylor group has developed a microfluidic organ‐on‐a‐chip microphysiological system called the vascularized liver acinus microphysiological system^72^ (Figure 2e). They are one of the few groups to incorporate tissue specific vasculature into their model. They introduced liver sinusoidal ECs (LSECs) and found that they mimic their in vivo function by not exhibiting tight junctions and having large pore fenestrations. In addition, Li et al. have incorporated hepatic stellate cells (HSCs), MSCs, and LSECs into self‐organized liver buds.^73^

### 4.5 Skin

The skin is the interface between the organism and the physical world. It is responsible for many physiological functions such as thermoregulation, sensations, and regulating what can enter and exit our bodies. Topically applied drugs must permeate the epidermal barrier, whereas systemically delivered drugs are controlled by the endothelial barrier. Incorporating vasculature into bioengineered skin is essential for improving the lifespan, graftability, and for studying the systemic delivery of drugs from/to the skin.^95^

Coculturing ECs within the dermal compartment and stimulation with growth factors such as VEGF and FGF has been shown to stimulate capillary formation.^74^, ^75^, ^76^, ^96^ However, the vasculature that has been formed lack perfusion and therefore are not functional to study systemic delivery of drugs. Recently, Groeber et al. used a segment of decellularized porcine jejunum to form a reconstructed model and recellularized it with fibroblasts, keratinocytes, and human microvascular ECs.^77^ They then connected the vascular network that formed to a bioreactor perfusion system to mimic subcutaneous circulation.

Abaci et al. made a reconstructed model by utilizing a sacrificial layer of alginate channels to form vascular networks in the dermal compartment of skin constructs^78^ (Figure 2f). A physiologically relevant vascular structure was formed by using a 3D‐printing technique and ECs or iPSC‐derived ECs were then seeded into the channels that were formed initially by alginate after they were dissolved. In vitro and in vivo testing revealed permeability properties that are comparable to subcutaneous vasculature. This also allows better compatibility with integrating bioengineered skin into drug screening platforms.

### 4.6 Gut

The ability of the intestines to filter waste from the blood as well as absorb nutrients is critical for health. Any disturbing of that ability usually results in serious health complications. If there is damage to the intestine itself or to the vasculature, that can result in improper absorption of nutrients, as well as secretion of pathogenic bacteria into systemic circulation. Intestine transplants have high rates of complications due to a variety of reasons but especially because of higher immune suppression needed due to the innate immune system and antigen presenting capabilities of the gut making rejection more likely as well as too much suppression resulting in gut dysbiosis or infection.^80^

Current methods for bioengineering intestines include using decellularized scaffolds. In one model, intestines were harvested from rats and decellularized. The acellular scaffolds were then seeded with iPSC‐derived hind‐gut spheroids and HUVECs.^80^ The resulting engineered intestine was then transplanted into mice or cultured in vitro. Upon analysis and staining it was evident that the intestine was expressing intestine specific proteins and connecting to the host vasculature, with the transplanted intestines having greater expression rates than the in vitro cultured intestines.

Another model is the intestine on a chip model, which involves using intestinal organoids, harvested via biopsy from intestinal crypts.^81^ Using a porous PDMS chip with two parallel microchannels coated with ECM, the spheroids were seeded. One channel was designated epithelial and the other was designated vascular. The chip displayed proper intestinal cell polarity as well as the formation of tight junctions and adherens junctions and the formation of villus‐like structures. As this model used harvested intestinal microvasculature on the chip, there was tissue specific vasculature; however, there were no studies to see if the gene expression in the microvasculature gene expression changed over time to become less specific, which has occurred in other models.

### 4.7 Other organs

In skeletal muscle, vasculature does not only supply nutrients and remove waste, the pericytes associated with skeletal muscle vasculature also play a role in muscle development and growth. As shown in a study in mice, depleting the pericytes of mice results in muscle hypertrophy, indicating that pericytes have their own role and it is distinct from that of the satellite cells that are skeletal muscle cell precursors. Current vascularized skeletal muscle models include models generated from hiPSC in hydrogels. In one model, hiPSCs were cultured in a monolayer in a medium to commit to a PAX7+ cell lineage and then cultured in 3D with a differentiation medium.^86^ Constructs were created containing the differentiated cells, skeletal muscle pericytes, and motor neurons. This model exhibited muscle specific phenotype indicated by wholemount IF staining for myosin heavy chain myotubules and laminin‐positive ECM, which was confirmed by Western blot. The presence of vasculature was confirmed with IF Isolectin staining. Another model used reconstructed tissue from mice cells.^87^ The muscles were harvested and the cells were enriched for myoblasts and cultured in a monolayer. Differentiation was induced with medium and the cells were delaminated, collected, and pinned. After being pinned, the monolayer organized and formed cylindrical structures. There were no assays for the mouse model to establish tissue specificity of the vasculature but there was successful engraftment into other mice.

Kidney vasculature plays a critical role in the filtration function of the kidney. A cell type closely associated with the vasculature is podocytes, which are needed for filtration to occur in the glomeruli. Podocyte depletion can result in proteinuria and glomerulosclerosis. One vascularized kidney model used a mixed population of embryonic kidney cells isolated from the glomerulus, and made a kidney bud via condensation.^82^ After condensing, the bud possessed microcirculation and resembled the original organ tissue. When using mixed adult kidney cells, the resulting bud had poorer organization and bared little to no resemblance to the original tissue. Another model used hiPSC to create podocytes and nephron progenitor cells, which were then used to form spheroids.^83^ Grafting the podocytes and nephron progenitor spheres along with HUVECs, and MSCs into mice resulted in preferential clustering of the de novo vasculature within the graft to the podocytes. Analysis performed of the hiPSC‐derived podocytes showed that the podocytes demonstrate polarity and also that the gene expression is similar to that of human podocytes, with 190 of 300 genes overlapping. The mixed kidney buds were not examined for tissue‐specific vascular phenotype.

The main function of the pancreas involves glucose sensing from the blood through highly vascularized pancreatic islets. When pancreatic islets are cultured ex vivo, there is a significant loss in the EC populations, with >50% of the ECs being lost by Day 2 of culture, and almost all of the ECs being lost by Day 4 of culture.^84^ There have been a couple of vascularized models that aimed to avoid this complication. One microfluidic model used ex vivo transplanted islets and a nozzle device to reduce shear stress. This model seems to result in EC retention and maintains the glucose stimulated Ca^2+^ emissions.^84^ Another model involves harvesting islets and combining them with HUVECs, adipose‐derived MSCs, and collagen.^85^ The mixture is then cast and cultured for 3 days and then transplanted into diabetic mice. The mice who received the transplants were able to return to normoglycemia. While no studies were done to examine pancreatic specific vasculature markers, there were studies that showed that the transplanted islets were highly vascularized once harvested.

The vasculature in the lungs is among the most complex in the body with systems for pulmonary and bronchial circulation. The pulmonary circulation system contains many specialized vessels directly connected to the heart, whereas the bronchial circulation system has a very elaborate microvascular network that is combined with bronchioli and alveoli. Airway organoids have been made through self‐assembly by Tan et al. by combining human primary bronchial epithelial cells, lung fibroblasts, and lung microvascular ECs^79^ (Figure 2g). They found YAP is also necessary for the development of the organoid, similar to in vivo development. The organoids also displayed proximal and distal epithelial markers, showing potential to be physiologically relevant.

Tumor blood vessels are critical to the supply of nutrients to the tumor, as well as dissemination of the tumor cells for metastasis. Tumor vasculature is often abnormal with a leaky structure and fast growing as well as irregularly branched with varying perfusion.^97^ There is a greater number of vessels in between tumor and host vasculature then at the center of the tumor. One class of tumor vessels has significantly fewer pericytes than the comparable healthy vessels.^98^ Tumor vessels that are surrounded by more pericytes have irregular ordering. The BM of another class of tumor vessels is abnormally thick with multiple layers, and multiple classes of tumor vasculature contain enlarged and disorganized smooth muscle cells. Current tumor models include ex vivo dissected tumors. Using human carcinoma lines grafted into mice and tumor tissue collected from patients, the tumor samples were embedded into a microfluidic chip with five channels.^89^ Attempts were made to get large sections of the tumors to allow for better mimicry of the natural nutrient and waste gradients. The tumors were embedded into agarose and seeded into the channels for treatment with chemotherapy drugs. Another model involved using spheroids.^88^ The spheroids were comprised of human ECs and human tumor cells, which were formed using nonadherent round bottom plates. The spheroids were embedded into fibrin gel and incubated. After 24 hr of incubation, vessel sprout structures were observed and by day three, the spheroids reorganized and infiltrated further into the center of the spheroid connecting the sprouting vessels. A landmark perfusable in vitro vascularized tumor model was established in Sobrino et al.^90^ Using a microfluidic platform and seeding it with ECs and ECM, vessel fragments formed in the first 2–3 days and a complete network was visible around Days 5–7. Tumor cells were then added to the platform and after 6 days formed spheroids close to the vasculature with vessels infiltrating through the spheroids as well as surrounding the spheroids. The formation of microvasculature was also observed. Although the models demonstrated vasculature, there was no analysis of the vasculature to see if it matched any of the structural or molecular characteristics associated with tumor vasculature.

## 5 CONCLUSIONS

The characterization of tissue‐specific vasculature and bioengineering of vascularized organ models are two interdependently advancing areas of research. One major step that is yet to be taken for vascularized organ models is to further explore whether the tissue‐specific vascular markers are expressed and functional properties are represented in these models. This will provide more confidence in the tissue‐specificity of engineered vasculature and a more reliable platform to further dissect the roles of different microenvironmental cues and expand our current knowledge in vascular diversity.

In the majority of literature regarding vascularized tissue models, HUVECs have been the choice of EC type, partially based on the vast number of preceding publications showing the capability of these cells to spontaneously form vasculature in vitro. Although, HUVECs do not represent an ideal cell type to create tissue‐specific vasculature, some studies interestingly indicate that HUVECs can take on the specific vasculature phenotype for the organ. For example, in a study from Kamm's group,^99^ HUVECs established an intact BBB with tight junctions when they were cocultured with rat astrocytes and neurons, suggesting the determining role of the microenvironment and the potential existence of endothelial plasticity. As research moves forward in developing more realistic tissue models, endothelial plasticity is something that could be of significance for practical applications.

With the development of organ models, multiple organs/tissues have been successfully integrated onto microfluidic culture platforms for human‐relevant and patient‐specific drug testing.^100^ In some of the platforms, individual tissue chambers were connected to each other through microchannels that are coated with ECs. These multiple‐organ (a.k.a. human chip) systems may also allow for understanding the tissue‐specific characteristics of vasculature by examining the phenotype and function of ECs in proximity to each tissue chamber.^101^

While incorporating vasculature into tissue models is becoming more common, most disease models in the literature are not vascularized. Several disease models have been developed using iPSCs derived from patients. These include, but are not limited to, Alzheimer's disease,^102^ cystic fibrosis in the pancreas,^103^ and gastric *Helicobacter pylori* infection^104^ models using self‐assembled iPSC‐derived tissues. As our understanding of tissue specific vasculature increases, the methods described in this article to engineer vasculature for organ models can be further adapted to incorporate vasculature in the organ‐specific disease models to elucidate vascular mechanisms, discover drug candidates, and examine systemic drug delivery.

The lymphatic system works synergistically with the blood microvasculature in maintaining the various organs in the body and is one of the vasculature components that should be included in tissue‐engineered models in the future.^105^ Although the lymphatic vasculature has not been the primary focus of this review, many of the models and techniques shown here for blood microvasculature formation may also be used for lymphatic vasculature. However, additional modifications, such as changing the type of matrices, may need to be made when incorporating lymphatic ECs in tissue‐engineered models.^106^

Although the methods to include shear stress, cyclic stretch as well as other vascular cell types such as smooth muscle cells have been established, these factors are typically not represented in majority of vascularized organ models understandably due to already existing complexity of these models. In such models, it becomes challenging to incorporate and coculture large numbers of different cell types. Other bottom‐up approaches like iPSC‐derived organoids discussed above may offer a solution to this problem. However, in iPSC‐derived organoids, generation and spatial control of tissue subcomponents consisting of multiple lineages is still a prevailing challenge yet to be addressed.^107^

In light of the growing body of evidence on the vascular heterogeneity in different organs, complete understanding of tissue‐specific vasculature will have an overwhelming positive impact on targeted delivery of drugs without off‐target side effects and reversing the high attrition rates of drug candidates which otherwise would offer effective therapies for patients. The pioneering studies reviewed in this article on in vitro models of vascularized organs will unequivocally be pivotal for the understanding and medical utilization of the tissue‐specific vasculature.
